# Cardiovascular response to NO donors in mice deficient in NO-GC

**DOI:** 10.1186/2050-6511-14-S1-P27

**Published:** 2013-08-29

**Authors:** Dieter Groneberg, Anna Schlembach, Andreas Friebe

**Affiliations:** 1 Physiologisches Institut, Universität Würzburg, Würzburg, Germany

## 

The NO/cGMP cascade is essential for control of the cardiovascular system and it is also involved in the pathophysiology of several cardiovascular diseases. One of the main vascular factors involved in the relaxation of blood vessels and regulation of blood pressure is nitric oxide (NO). Target of NO is the NO-sensitive guanylyl cyclase (NO-GC) which is expressed in different cell types in the cardiovascular system (e.g. smooth muscle cells, cardiomyocytes). The stimulation of the NO/cGMP cascade is used pharmacologically for the treatment of coronary heart disease; substances commonly used are compounds such as glycerol trinitrate (NTG) or isosorbide dinitrate which are thought to act by increasing NO concentration and activation of NO-GC.

We have previously shown that complete knockout of NO-GC (GCKO) in mice led to an increase in systolic blood pressure (+30 mmHg) concomitant with an abolished NO responsiveness of vascular smooth muscle. Smooth muscle-specific KO mice for NO-GC (SM-GCKO) showed an increase in systolic blood pressure identical to that seen in general GCKO mice.

In order to find out the relative contribution of different cell types expressing NO-GC in the cardiovascular system, we here used catheter measurements to compare the effect of different NO donors (NTG and DEA-NO) on blood pressure and heart rate *in vivo*. In WT mice, both NTG and the radical NO donor DEA-NO reduced systolic blood pressure and increased heart rate indicating the contribution of the baroreceptor reflex. The deletion of NO-GC in mice (GCKO) abrogated both effects. We are currently investigating mice lacking NO-GC in smooth muscle cells, endothelial cells and cardiomyocytes.

